# Analysis and Optimization of Vertical NPN BJT for Strong Magnetic Fields

**DOI:** 10.3390/mi16060671

**Published:** 2025-05-31

**Authors:** Xinfang Liao, Kexin Guo, Changqing Xu, Yi Liu, Fanxin Meng, Junyi Zhou, Rui Ding, Juxiang Li, Kai Huang, Yintang Yang

**Affiliations:** 1 Guangzhou Institute of Technology, Xidian University, Guangzhou 510555, China; xinfangliao_xidian@163.com (X.L.); guokexin_gkx@163.com (K.G.); 24181214454@stu.xidian.edu.cn (J.Z.); 2 Laboratory of Digital IC and Space Application, School of Microelectronics, Xidian University, Xi’an 710071, China; fxmeng@stu.xidian.edu.cn (F.M.); ytyang@xidian.edu.cn (Y.Y.); 3 The Fifth Electronics Research Institute of the Ministry of Industry and Information Technology, Guangzhou 511370, China; dr_5261@163.com; 4 Northwest Institute of Mechanical and Electrical Engineering, No. 5, Biyuan East Road, Xianyang 712099, China; lijielijuxiang@163.com (J.L.); hoko21@sina.com (K.H.)

**Keywords:** BJT, magnetic field, asymmetry, current gain

## Abstract

This study systematically investigates the electrical characteristics of the vertical NPN bipolar junction transistor (VNPN BJT) in the strong magnetic field environment, focusing on analyzing the effects of magnetic field direction and intensity on key parameters such as terminal current and current gain (*β*). The simulation results show that the magnetic field induces changes in the carrier distribution, thereby affecting the current transport path. Through the in-depth analysis of electron motion trajectories, potential distribution, and Hall voltage, this paper reveals the physical mechanisms behind the device’s characteristic changes under the magnetic field and discovers that the inherent asymmetry of the BJT structure induces significant magnetic anisotropy effects. On this basis, a design for interference-resistant structures in strong magnetic field environments is proposed, effectively suppressing the adverse effects of magnetic-field-sensitive directions on BJT performance and significantly improving the device’s stability in complex magnetic field environments.

## 1. Introduction

In strong magnetic field environments, the transport behavior of charge carriers within semiconductors can be profoundly altered. Phenomena such as current path reconfiguration, carrier mobility degradation, and voltage drift emerge, leading to significant variations in device parameters and functional reliability. In lightly doped silicon, the magnetic field alters the space-charge regions, resulting in a pronounced magnetoresistive effect [1,2].

Experimental investigations have confirmed the presence of such magnetoresistive phenomena in a range of semiconductor devices, including p–n junctions, MOSFETs, and JFETs [3,4,5,6]. Moreover, optoelectronic components such as avalanche photodiodes and silicon-based light-emitting diodes exhibit notable variations in performance when subjected to high magnetic fields, reflecting the broader influence of magnetic environments on both electronic and photonic functionalities [7,8,9]. Therefore, studying the behavior of semiconductor devices under strong magnetic fields is of both significant theoretical interest and practical engineering value for device design and application in high-magnetic-field environments. For bipolar junction transistors (BJTs), their behavior under a magnetic field is more complex due to the presence of multiple PN junctions, exhibiting strong directional and nonlinear characteristics.

This paper addresses the above issues by establishing a vertical NPN BJT model using the Sentaurus TCAD simulation platform (Version 0-2018.06-SP2). We analyze the effects of different magnetic field strengths on the device’s electrical behavior in two typical magnetic field directions: perpendicular to both the device surface and the main current direction. The research objectives include the following: systematically evaluating the impact of magnetic field on the BJT’s output characteristics, current gain, as well as the variations in collector, base, and emitter currents; revealing the carrier path deviation induced by the magnetic field, Hall voltage formation, potential distribution changes, and depletion region expansion; constructing and explaining the physical mechanisms of performance degradation in BJTs under magnetic fields; and providing a theoretical basis for the future optimization and anti-magnetic design of BJT structures in magnetic field environments.

## 2. Model and Methods

**Figure 1:**
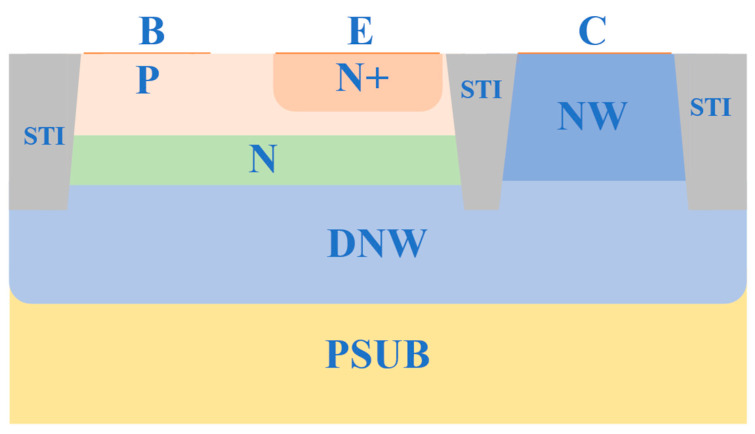
Structure of the standard vertical NPN BJT device.

This paper models a standard silicon-based VNPN structure, as illustrated in Figure 1.

The main mobility models for semiconductor carriers include lattice vibration scattering, ionized impurity scattering, and Coulomb scattering between carriers. These three types of scattering are interrelated, and their contribution to total mobility is defined as $\mu_{1}$. After an external magnetic field is applied, carriers are scattered by the magnetic field, further altering their mobility. The mobility induced by the magnetic field is referred to as Hall mobility $\mu_{hall}$. According to the Hall effect model, the relationship between conductivity σ and Hall mobility is given by the following [10]:

(1) $$\mu_{hall}=\left|R_{H}\sigma \right|$$

where $R_{H}$ represents the Hall coefficient. The total mobility considering the magnetic field effect is given by the following:

(2) $$\mu =\frac{\mu_{I}\mu_{hall}}{\mu_{I}+\mu_{hall}}$$

Under a steady-state magnetic field, the transport behavior of electrons and holes is influenced by the Lorentz force, causing their effective mobility to change, which in turn affects the current density. The corrected expressions for electron and hole current densities are given by the following [11]:

(3) $${J_{n}}^{*}=\frac{1}{1+{\left({\mu_{n}}^{*}B\right)}^{2}}[(q\mu_{n}nE)+(qD_{n}\nabla n)+{\mu_{n}}^{*}B\times (q\mu_{n}nE+qD_{n}\nabla n)]$$

(4) $${J_{p}}^{*}=\frac{1}{1+{\left({\mu_{p}}^{*}B\right)}^{2}}[(q\mu_{p}pE)+(qD_{p}\nabla p)+{\mu_{p}}^{*}B\times (q\mu_{p}pE+qD_{p}\nabla p)]$$

where $J_{*}$ denotes the current density contributed by either electrons or holes, while $\mu_{*}$ represents the corresponding mobility. *q* is the elementary charge and n and p are the electron and hole concentrations, respectively. The electric field strength is denoted by E, and the magnetic flux density is denoted by B, and both are treated as vectors. Additionally, $D_{*}$ stands for the diffusion coefficient associated with the respective carriers.

These equations indicate that when a magnetic field B is applied perpendicular to the current direction, the densities of electrons and holes change. These changes affect the distribution of current density and the transport mechanism [12,13,14,15]. Therefore, under the influence of the magnetic field, the conductive characteristics of the BJT must be recalculated and analyzed. This is carried out based on the corrected mobility and current density equations shown in Formulas (3) and (4), which describe the working state of the PN junction device in the magnetic field.

**Figure 2:**
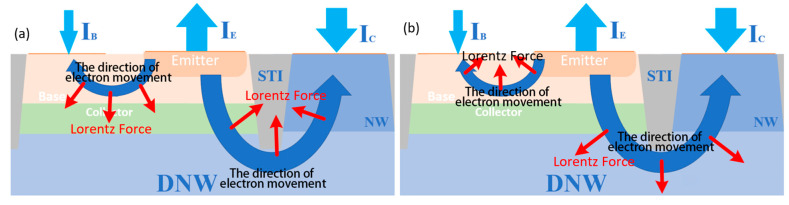
The direction of electron motion in the BJT under the influence of the Lorentz force for (**a**) positive Z-direction magnetic field and (**b**) negative Z-direction magnetic field, respectively.

In this study, the applied magnetic field is directed perpendicular to the modeled 2D plane. The direction pointing outward from the plane is defined as the positive Z-direction, while the direction pointing inward is defined as the negative Z-direction. The effect of the magnetic field on carrier mobility is mainly reflected in the shift in their motion direction, which can be analyzed based on the direction of the Lorentz force. According to the previous theoretical analysis and the Lorentz force expression F=qv×B, the trajectories of the carriers are altered under the magnetic field, thereby modifying the overall current transport direction. These trajectory shifts under both positive and negative Z-direction magnetic fields are illustrated in Figure 2, which depicts the motion of electrons in the BJT influenced by the Lorentz force.

## 3. Results and Discussion

### 3.1. The Change in Carrier Density Inside the Device

**Figure 3:**
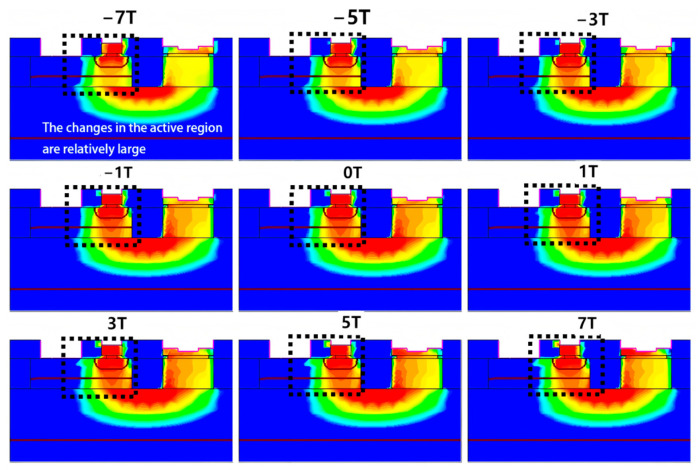
Current density distributions of the BJT under the magnetic field with different directions and strengths.

The main impact of the magnetic field on a BJT occurs in the active region, where it alters the direction of carrier motion, thereby changing the carrier distribution. As shown in Figure 3, the current density in the active region exhibits the most significant variation under the magnetic field. The framed region is the active area, where the current density changes most significantly under the magnetic field. A clear reduction in the current density is observed as the magnetic field strength increases, with a more pronounced decrease under the negative Z-direction magnetic field. In contrast, for the carriers in the buried layer and deep well, the change in current density due to the magnetic field is minimal.

#### 3.1.1. Change in Collector Current Formation

For the collector current, the relationship to be followed is as follows:

(5) $$I_{C}=I_{nc}+I_{rb}+I_{CBO}$$

where $I_{nc}$ represents the main current formed by electrons flowing through the collector region, $I_{rb}$ is the recombination current in the base region, and $I_{CBO}$ is the reverse saturation current. The reverse saturation current $I_{CBO}$ is a small current generated by the reverse bias of the PN junction between the collector and the base. Its value is usually very small and can typically be neglected. In practical cases, the collector current $I_{C}$ is mainly contributed by $I_{nc}$, so in the analysis of collector current, $I_{rb}$ and $I_{CBO}$ can be neglected, and only the dominant role of $I_{nc}$ in the overall output current is discussed.

**Figure 4:**
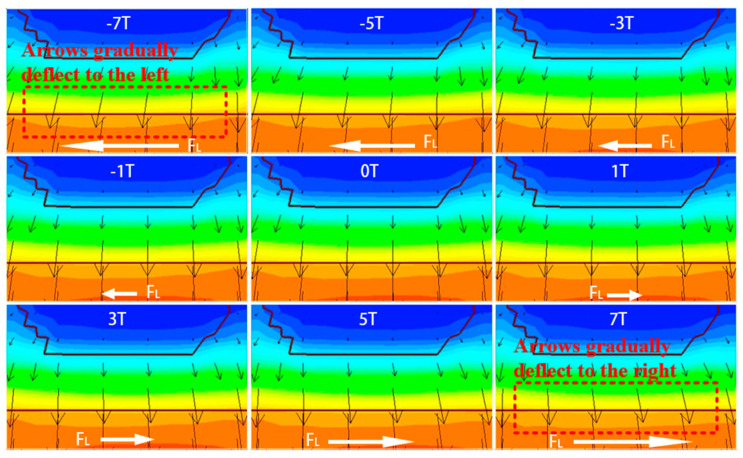
Velocity vectors of electron motion in the active region under different magnetic field directions and strengths.

**Figure 5:**
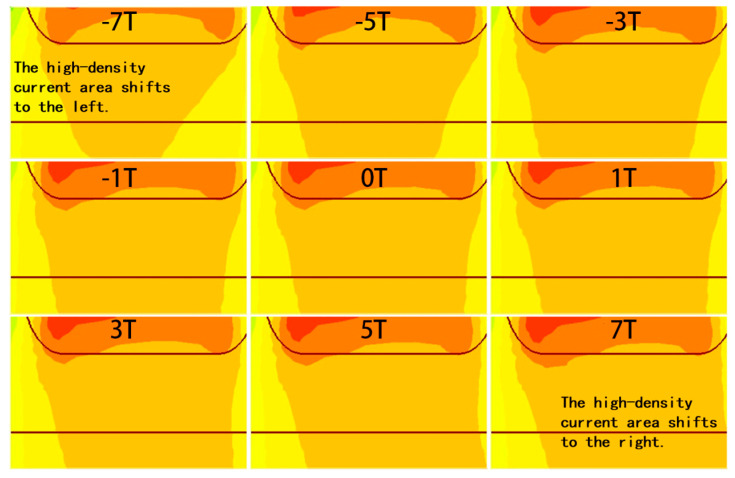
Variation in $I_{nc}$ in the active region under magnetic fields from −7 T to 7 T.

When a magnetic field along the negative Z-direction is applied, the electrons forming the collector current $I_{nc}$ in the active region shift to the left, causing an overall leftward shift in the carriers. The degree of this shift becomes more significant as the magnetic field strength increases. Although the total mobility remains unchanged, the transverse mobility increases relative to the longitudinal mobility, which decreases. Given that the primary direction of carrier movement in the active region of a VNPN device is longitudinal, the decrease in longitudinal mobility directly leads to a reduction in the effective mobility, which in turn causes a decrease in the collector output current $I_{C}$. In Figure 4, the black arrows represent the velocity vectors of electrons, where the direction indicates the motion direction and the length reflects the motion velocity. The white arrows indicate the direction of the Lorentz force acting on the electrons. It is observed that the magnetic field causes a noticeable deflection in the electron trajectories but has little effect on their absolute velocity. This suggests that the magnetic field mainly alters the direction of carrier motion rather than significantly changing their mobility. In Figure 5, under the negative Z-direction magnetic field, I_nc_ shifts leftward, while under the positive Z-direction magnetic field, it shifts rightward. As shown in Figure 4 and Figure 5, the application of a negative Z-direction magnetic field leads to a clear leftward shift in electron motion direction and a corresponding leftward deviation in current density within the active region. 

**Figure 6:**
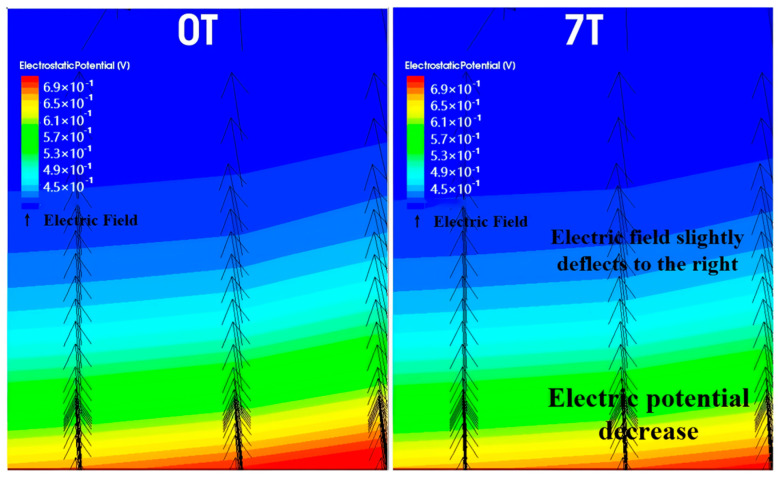
Electron accumulation on the right side due to the positive Z-direction magnetic field, creating a Hall voltage and lowering the potential on the right side of the active region.

In contrast, when a magnetic field along the positive Z-direction is applied, the electron movement direction shifts to the right, causing the carriers in the active region to also shift rightward, leading to a similar decrease in effective mobility. However, due to the structure of the VNPN device, the active region’s right side is adjacent to the shallow trench isolation (STI) region. During the transverse shift, the electrons encounter this insulating structure, and their motion is halted. As a result, electrons accumulate at the boundary between the active region and the STI region. This accumulation of electrons further induces the establishment of a Hall voltage, which compensates, to some extent, for the carrier displacement caused by the magnetic field. Due to this mechanism, the effect of a positive Z-direction magnetic field on the collector current is noticeably weaker than that of a negative Z-direction magnetic field, which means that the decrease in $I_{C}$ is less under the positive Z-direction magnetic field. Figure 4 and Figure 5 also illustrate the rightward shift in electron motion direction and the corresponding drift of current density toward the STI boundary under the positive magnetic field. As shown in Figure 6, under the positive Z-direction magnetic field, there is a clear accumulation of electrons at the right-hand boundary, which leads to a local reduction in potential. In addition, the electric field within the active region slightly tilts to the right, indicating that the Hall voltage has been superimposed on the original internal electric field.

Under the condition that the voltages at the three device terminals remain equal, applying an external magnetic field usually leads to a decrease in the collector current $I_{C}$, with the current decay becoming more pronounced as the magnetic field strength increases. Overall, the suppression effect of the negative Z-direction magnetic field on the device current is significantly stronger than that of the positive Z-direction magnetic field. When the magnetic field strength is −7 T, the collector current decreases by the maximum amount, reaching 5.52%.

**Figure 7:**
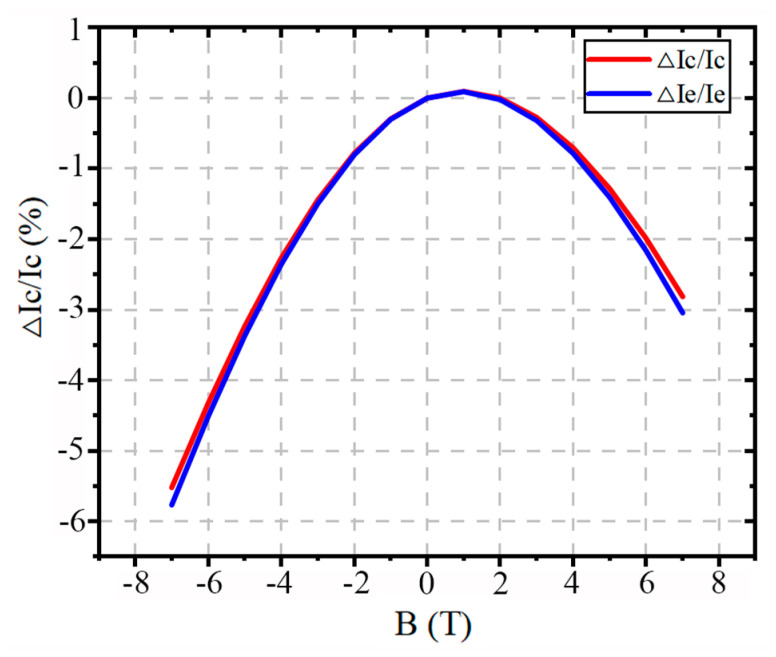
Collector and emitter currents generally show a decreasing trend as the magnetic field strength increases, with greater suppression under the negative Z-direction magnetic field.

Since the emitter current $I_{E}$ is typically approximated as equal to the collector current, the trends of the two currents are highly consistent, as shown in Figure 7. As $I_{C}$ decreases, the emitter current also shows a similar decreasing behavior. It is worth noting that the current does not decrease in the range of 0–2 T; instead, a slight increase is observed. This suggests that a weak magnetic field in the positive Z-direction may facilitate current transport by reducing the carrier drift distance. At the magnetic field of −7 T, the decrease in $I_{E}$ reaches its maximum value of 5.77%. These results further confirm the significant impact of the magnetic field, especially the negative Z-direction magnetic field, on the current transport characteristics of the device.

#### 3.1.2. Change in Base Current Formation

**Figure 8:**
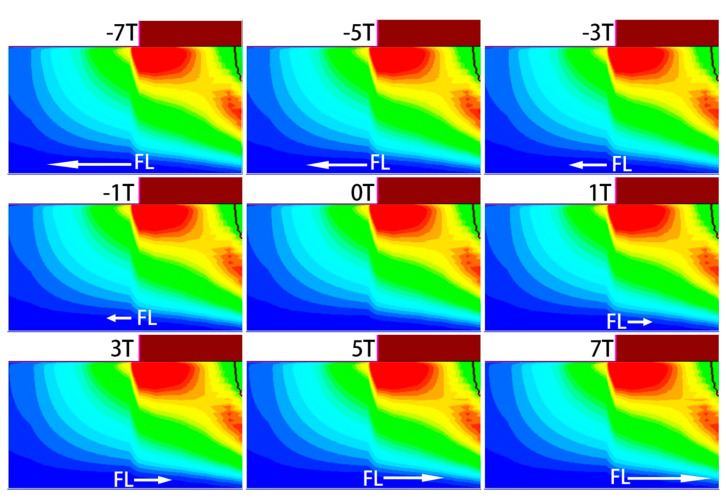
The effect of the magnetic field on the hole current in the base region is minimal.

For the base current, ignoring the reverse saturation current $I_{CBO}$, the relationship to follow is as demonstrated below:

(6) $$I_{B}=I_{pe}+I_{rb}$$

where the base hole drift current $I_{pe}$ represents the current formed by holes drifting across the emitter junction. According to the mobility expression under the magnetic field, the effective carrier mobility in a magnetic field is inversely proportional to the square of the mobility under the zero magnetic field condition. Therefore, the greater the mobility under the zero magnetic field, the more significant the change in mobility caused by the magnetic field. Since the intrinsic mobility of holes is about one-fourth that of electrons, the change in mobility under the magnetic field is relatively small. In the device simulation of this study, it was observed that even under a strong magnetic field of up to 7 T, the change in the hole current density remains extremely weak. Hence, when analyzing the magnetic field’s effect on the base current, the hole drift current $I_{pe}$ can be considered almost unchanged. As shown in Figure 8, the spatial distribution of the hole current density in the base region exhibits no significant change under the influence of the magnetic field.

The recombination current $I_{rb}$ is analyzed based on the recombination rate $R_{v}$ and the effective area of recombination. This analysis focuses only on non-radiative recombination in the BJT, including SRH recombination and Auger recombination.

The relationship between recombination current density and recombination rate is usually given by the following:

(7) $$J_{rec}=q\cdot R_{v}\cdot A$$

where $R_{v}$ is the recombination rate and A is the effective recombination area.

**Figure 9:**
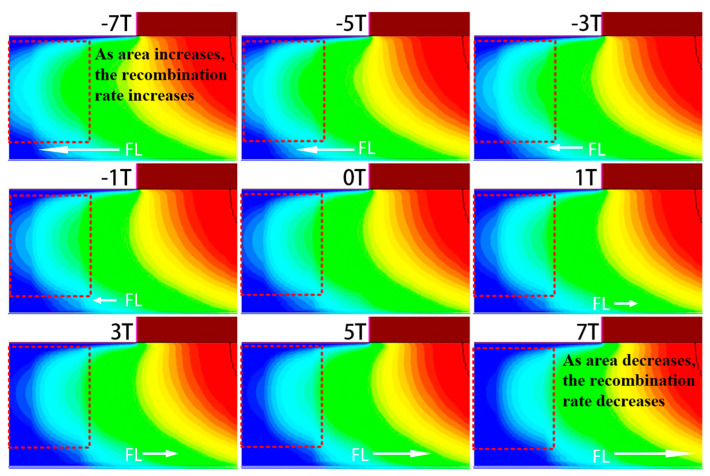
Change in SRH recombination rate $R_{SRH}$.

**Figure 10:**
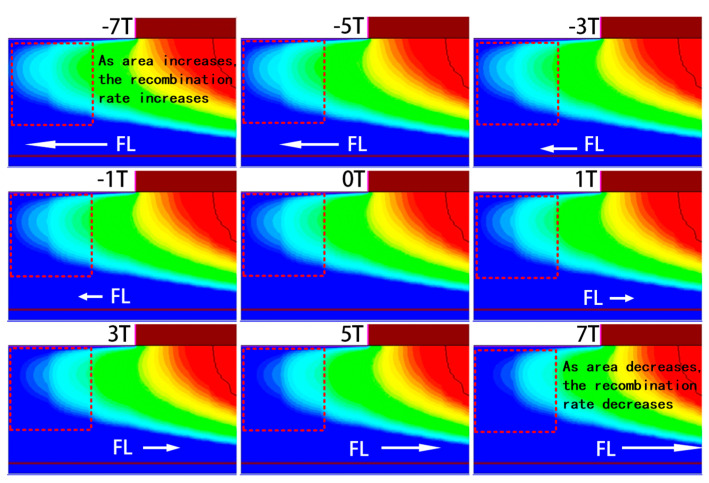
Change in Auger recombination rate $R_{Auger}$.

Both the SRH recombination rate $R_{SRH}$ and Auger recombination rate $R_{Auger}$ show the same trend under the magnetic field. As illustrated in Figure 9 and Figure 10, which show the spatial distributions of SRH and Auger recombination rates, respectively, the recombination rate and recombination area both decrease to some extent under a positive Z-direction magnetic field, while both increase significantly under a negative Z-direction field. In Figure 9 and Figure 10, the positive Z-direction magnetic field reduces the recombination area and rate, while the negative Z-direction magnetic field increases both. The change in the recombination area is closely related to the redistribution of carriers in the base region. The magnetic field influences electron trajectories, altering the recombination behavior in the base region. Under the negative Z-direction magnetic field, electrons in the base region are more likely to recombine, leading to an increase in the recombination current $J_{rec}$. In contrast, under the positive Z-direction magnetic field, the recombination process is somewhat suppressed, resulting in a decrease in $J_{rec}$.

**Figure 11:**
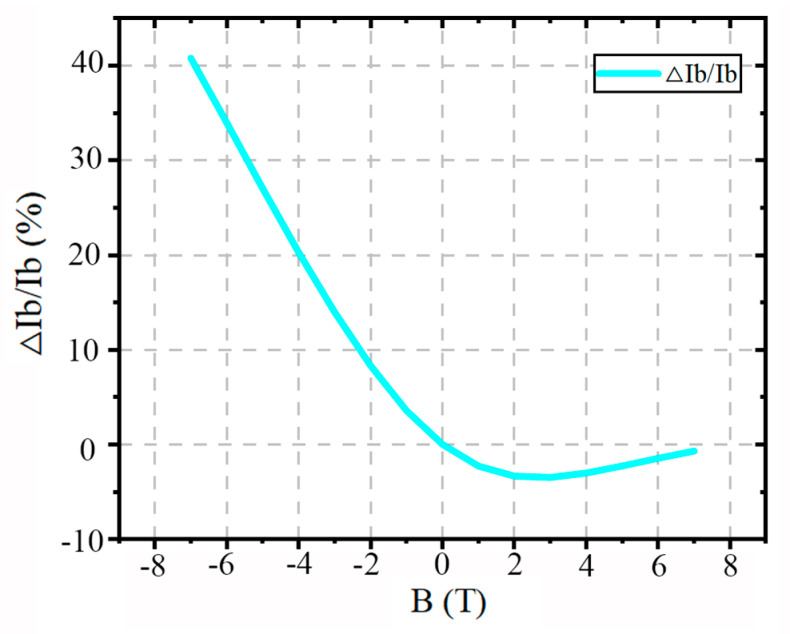
The base current increases significantly under the negative Z-direction magnetic field, while it decreases under the positive Z-direction magnetic field.

Therefore, for the base current, as the magnetic field strength increases, the base current also increases. As shown in Figure 11, the base current increases significantly under the negative Z-direction magnetic field, while it decreases under the positive Z-direction field. In the TCAD simulation, the base current reaches its maximum at 7 T in the negative Z-direction, increasing by more than 40%. Contrastively, in the positive Z-direction, the base current decreases by up to 4.2% with a magnetic field strength of 7 T. This result further demonstrates that the negative Z-direction magnetic field has a stronger enhancing effect on the base current.

### 3.2. Variation in BJT Current Gain

The Z-direction magnetic field changes the recombination behavior in the base region and generally suppresses the collector current, resulting in a variation in the BJT’s current gain. Based on the expressions for collector and base currents, the current gain of the BJT can be defined as follows:

(8) $$\beta =\frac{I_{nc}}{I_{pe}+I_{rb}}$$

When a negative Z-direction magnetic field is applied, the dominant collector current $I_{nc}$ decreases with the increasing magnetic field strength, while the base recombination current $I_{rb}$ increases significantly. The hole current $I_{pe}$ which is less sensitive to changes in the magnetic field, remains almost constant. Therefore, under a negative Z-direction magnetic field, the current gain of the BJT decreases. In contrast, under a positive Z-direction magnetic field, although $I_{nc}$ also decreases with increasing magnetic field strength, $I_{rb}$ shows a slight decrease, with the change being smaller than that of $I_{nc}$. The slight reduction in the base current partly compensates for the decrease in collector current, so while the current gain still decreases under the positive Z-direction magnetic field, the reduction is less pronounced compared to that of the negative Z-direction magnetic field. It is worth noting that in the range of 0–2 T under the positive Z-direction magnetic field, a slight increase in $I_{c}$ is observed, resulting in a minor enhancement in the current gain *β* around the magnetic field strength of 2 T.

**Figure 12:**
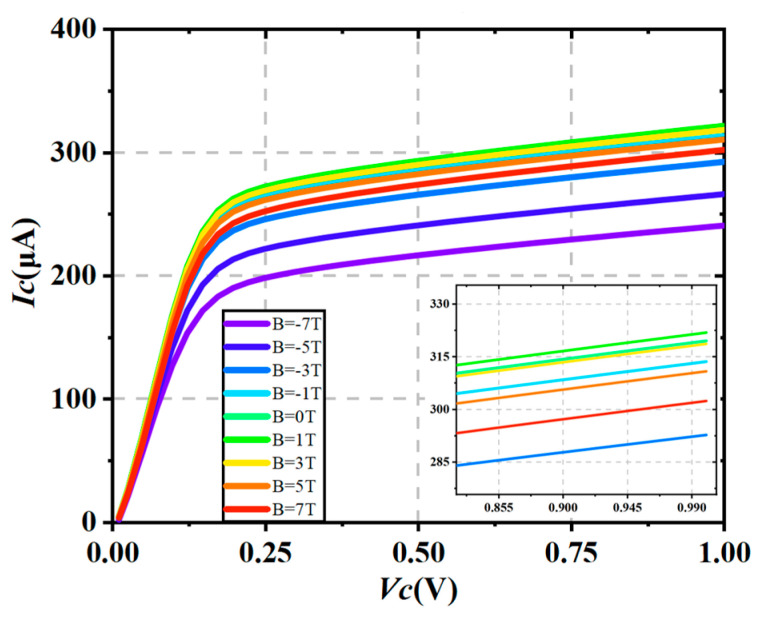
Output characteristics of the BJT under the magnetic field ranging from −7 T to +7 T.

**Figure 13:**
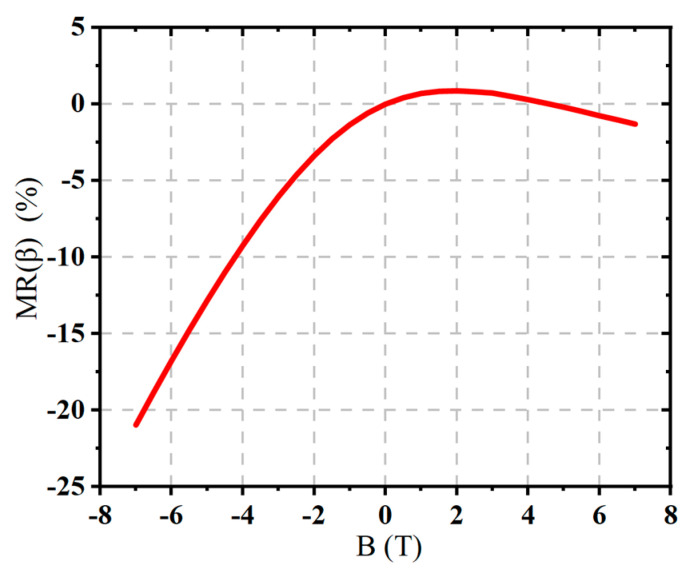
$MR\left(\beta \right)\;as\;a\;funtion\;of\;the\;magnetic\;field\;B\;for\;the\;BJT.$ The current gain decreases overall as the magnetic field strength increases, with a significantly larger drop under the negative Z-direction magnetic field.

As shown in Figure 12, from the simulation, it is observed that as the magnetic field strength increases, the output current of the device gradually decreases. As the magnetic field strength increases, the output current decreases overall, with the negative Z-direction magnetic field causing a more significant suppression effect compared to the positive Z-direction magnetic field. This highlights the anisotropy of the device’s response to the magnetic field. This trend is particularly evident under the negative Z-direction magnetic field, and its magnitude is significantly greater than that observed under the positive Z-direction field. By further plotting the relationship between current gain *β* and magnetic field, with the *β* value at a base current of $I_{B}$ = 10 μA as a representative indicator, the simulation results show that *β* generally continues to decrease with the increasing magnetic field strength, with a more significant drop under the negative Z-direction magnetic field. To provide a more intuitive depiction of the magnetic field’s effect on the device’s amplification ability, a normalized metric $MR\left(\beta \right)$ is introduced. The normalization formula is as follows:

(9) $$MR\left(\beta \right)=\left(\frac{\beta_{B}-\beta_{0}}{\beta_{0}}\right)\times 100\%$$

where $MR\left(\beta \right)$ represents the change in the current gain *β* under the magnetic field, $\beta_{B}$ is the current gain under different magnetic field conditions, and $\beta_{0}$ is the initial value of *β* without the magnetic field. This formula shows the variation in β under different magnetic field conditions, and based on this formula, we further plot the relationship curve of $MR\left(\beta \right)$ with the magnetic field B. From Figure 13, it is clearly evident that the negative Z-direction magnetic field has the most significant effect on the current amplification ability of the BJT.

## 4. Structure Optimization

Based on the above analysis, the magnetic field direction that most significantly impacts the electrical performance of the BJT is the negative Z-direction. The main reason for this is the asymmetry present in the device structure. Under the positive Z-direction magnetic field, the shallow trench isolation (STI) region on the right side of the base effectively prevents carriers from further shifting to the right. However, in the negative Z-direction magnetic field, there is no corresponding structural barrier on the left side of the device, causing the carrier’s shift path to extend, thereby making the influence of the magnetic field more pronounced.

**Figure 14:**
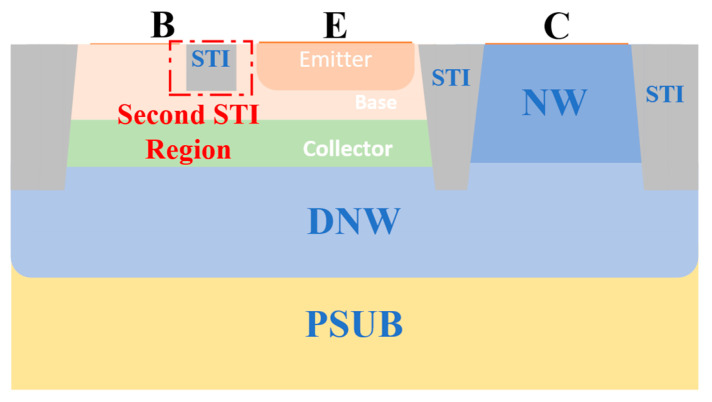
The optimized structure resistant to strong magnetic interference.

To address this asymmetry effect, this paper proposes a structural optimization scheme to enhance the stability of the device under strong magnetic fields. Specifically, on the traditional VNPN structure, an additional STI structure (referred to as the second STI region) is added to the left side of the emitter region and the right side of the base electrode, as shown in Figure 14. Its depth is similar to that of the BE junction but slightly smaller than the original STI depth, ensuring that the intervention on carriers is controlled and does not affect the normal conduction path.

**Figure 15:**
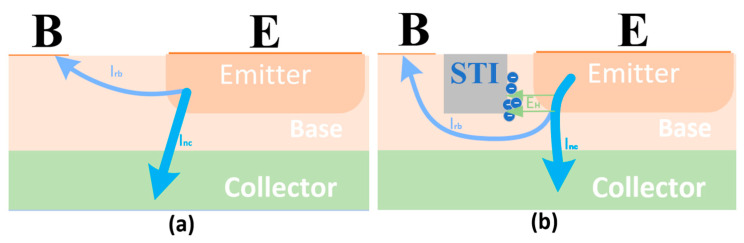
Motion paths of electrons in the base region under negative Z-direction magnetic field: (**a**) before optimization; (**b**) after optimization. Overall, the STI region accumulates carriers and generates a Hall field that induces a Lorentz force opposite to the magnetic deflection, thereby correcting the carrier trajectory. It also ensures that the recombination path of Irb remains extended regardless of the presence of a magnetic field, improving the current stability.

After the addition of the second STI region, the electron motion direction inside the BJT is illustrated in Figure 15.

The significant enhancement of the base recombination current $I_{rb}$ under the negative Z-direction magnetic field is the main factor leading to the decrease in current gain *β*. Therefore, the core goal of the optimization design is to suppress the increase in $I_{rb}$ induced by the negative Z-direction magnetic field and balance the shift behavior of the dominant current $I_{nc}$ under the magnetic field. The newly added second STI region can effectively suppress the transverse shift in carriers under the negative Z-direction magnetic field, thus hindering the shortening of the recombination path and reducing the loss due to the base recombination current. Additionally, this STI structure limits the excessive shift in $I_{nc}$, suppressing the increase in its path under the negative Z-direction magnetic field, which would otherwise reduce the effective mobility.

Furthermore, the spatial accumulation of electrons in the second STI region helps establish a Hall voltage. The electric field generated by this voltage has an opposite direction to the Lorentz force, thus partially counteracting the magnetic field’s driving effect on carrier displacement. This mechanism further stabilizes the transmission path of $I_{nc}$, effectively reducing the interference of the magnetic field on the transport of the dominant current.

**Figure 16:**
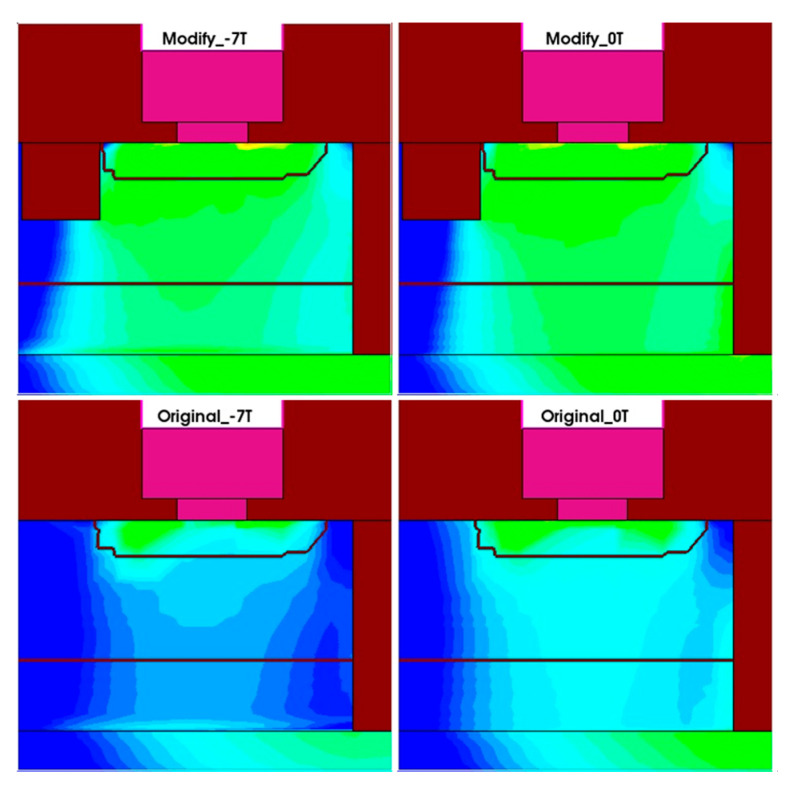
Comparison of electron density distributions in the active region under the negative Z-direction magnetic field and zero magnetic field for both the original and optimized structures.

Figure 16 shows the electron density distributions in the structures with and without the second STI region under both zero magnetic field and negative Z-direction magnetic field conditions. For the original BJT structure, under the negative Z-direction magnetic field, the main conductive region in the base experiences a decrease in the electron density due to the displacement of electrons. However, in the optimized structure, the second STI region blocks the further displacement of electrons, significantly slowing down the decrease in electron density under the negative Z-direction magnetic field, thus effectively reducing the impact of the negative Z-direction magnetic field on the BJT and improving the stability of the device in the strong magnetic environment. The optimized structure effectively mitigates the electron displacement caused by the magnetic field, resulting in a smaller reduction in the electron density and improved current stability.

**Figure 17:**
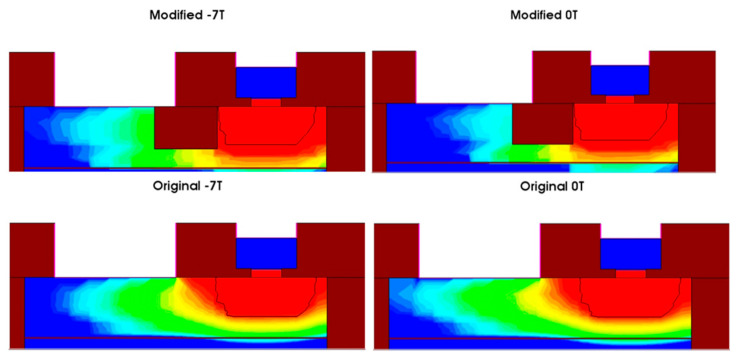
Comparison of recombination regions in the optimized and original BJT structures under negative Z-direction magnetic field and zero magnetic field.

Figure 17 shows the changes in the recombination regions in the structures with and without the second STI region under zero magnetic field and negative Z-direction magnetic field. The recombination region in the optimized structure is less affected by the magnetic field, indicating that Irb is also less influenced. Under the negative Z-direction magnetic field, the motion trajectory of electrons forming the recombination current $I_{rb}$ increases, causing the recombination region to expand, which leads to an increase in the base current and a subsequent decrease in the current gain. After introducing the second STI region, the effect of the negative Z-direction magnetic field on the recombination current and recombination region is suppressed, limiting the increase in the base current, thus effectively slowing down the decrease in the current gain.

**Figure 18:**
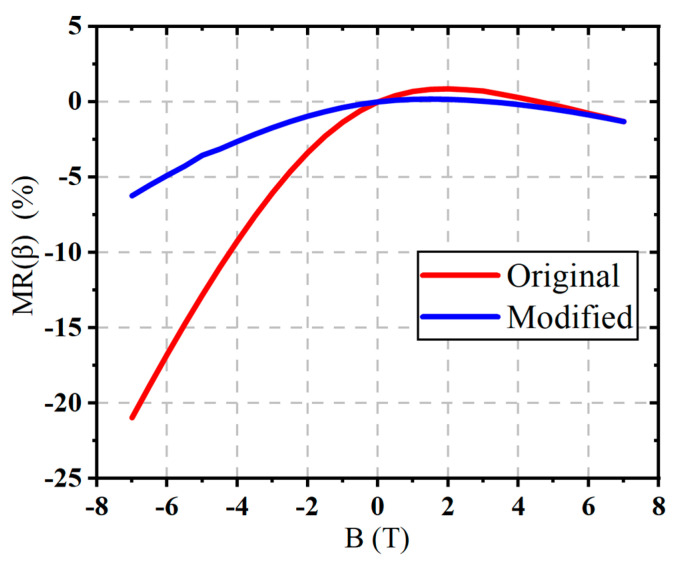
Comparison of MR(*β*) curves for the original and optimized BJT structures.

Figure 18 compares the MR(*β*) curves of the original and optimized BJT structures, with and without the second STI region. As shown in Figure 18, the improved structure significantly enhances the resistance to the negative Z-direction magnetic field. Under a 7 T magnetic field along the negative Z-direction, the $MR\left(\beta \right)$ of the original BJT is −42.9%, while the $MR\left(\beta \right)$ value of the optimized structure decreases to −4.61%. This represents a reduction of nearly 10 times, demonstrating the optimization effect of the second STI region on the BJT’s resistance to the strong magnetic interference.

## 5. Conclusions

This paper uses TCAD simulation to study the electrical characteristics of the vertical NPN bipolar junction transistor (VNPN BJT) under the strong magnetic field environment. The results show that the magnetic field induces carrier displacement and change in mobility, which in turn affects the collector current, base current, and current gain β. Among these, the effect of the negative Z-direction magnetic field is particularly significant, mainly due to the asymmetry in the device structure, leading to more severe carrier displacement.

To improve the device’s stability in strong magnetic environments, this paper proposes a structural optimization scheme by introducing a second STI region to the left of the emitter region and adjacent to the right side of the base electrode. This structure effectively suppresses the carrier displacement and base recombination behavior, significantly slowing down the decrease in β. The simulation results confirm that the optimized structure shows significantly enhanced interference resistance under the negative Z-direction magnetic field, demonstrating strong potential for engineering applications.

## Data Availability

The original contributions presented in the study are included in the article, further inquiries can be directed to the corresponding authors.
